# Low uptake of hypertension care after community hypertension screening events in Lagos, Nigeria

**DOI:** 10.1080/16549716.2018.1548006

**Published:** 2018-11-26

**Authors:** Heleen Elise Nelissen, Tochi Joy Okwor, Oluyemisi Khalidson, Akin Osibogun, Anja Helena Van’t Hoog

**Affiliations:** a Department of Global Health, Amsterdam UMC, University of Amsterdam, Amsterdam, The Netherlands; b Amsterdam Institute for Global Health and Development, Amsterdam, The Netherlands; c Centre for Epidemiology and Health Development, Lagos, Nigeria; d Department of Community Health, University of Nigeria Teaching Hospital Enugu, Enugu, Nigeria; e Department of Community Health, Lagos University Teaching Hospital, Lagos, Nigeria

**Keywords:** Recruitment, healthcare-seeking behavior, urban, private sector, low- and middle-income country, sub-Saharan Africa

## Abstract

**Background**: In Lagos, Nigeria, approximately 33% of the population suffers from hypertension, yet antihypertensive treatment coverage is low. To improve access to care, a decentralized pharmacy-based hypertension care model was piloted. This study reports on the recruitment strategies used and is part of a larger study to evaluate the feasibility of the care model.

**Objective**: To describe our experience executing three different strategies to recruit hypertensive patients in the program: community hypertension screenings, hospital and pharmacy referral.

**Methods**: Individuals with elevated blood pressure and no history of cardiovascular disease were referred to the program’s recruitment days to see a medical doctor for hypertension diagnosis and enrollment. Individuals were referred from community screenings, tertiary hospital outpatient clinics, and pharmacies participating in the program. For the community screenings, we report the number needed to screen (NNS) to find one individual with elevated blood pressure, the NNS to enroll one individual in the program, and factors associated with enrollment in the program among participants referred.

**Results**: We recruited 226 individuals (69%) in the program via the pharmacies, 97 (30%) via the community screenings, and 2 (<1%) via hospital referral. At the community screenings 3,204 individuals participated, 729 (23%) had elevated blood pressure and 618 (85%) were eligible for referral of whom 142 (23%) visited the recruitment days, and 97 (16%) enrolled. The NNS to find one individual with elevated blood pressure was 5, and the NNS to enroll one individual was 34. Enrollment in the program was associated with advancing age, blood pressure ≥160/100 and currently using antihypertensive medication.

**Conclusions**: Despite the potential attractiveness of community screenings to identify and refer individuals with hypertension, enrollment in the program was low. For future programs we recommend pharmacy referral as individuals seem more inclined to access care through healthcare providers they are familiar with.

## Background

In Lagos, Nigeria, around 33% of the population suffers from hypertension [1,2], which is higher than the country’s overall prevalence of 29% [3]. Hypertension is the main risk factor for cardiovascular disease (CVD) and associated morbidity and mortality [4]. Early identification of individuals with hypertension gives the opportunity to modify long-term risk through treatment, before serious complications occur [5]. Yet, in sub-Saharan Africa (SSA) awareness of hypertension is low and access to quality care is poor [6]. Consequently, antihypertensive treatment coverage and hypertension control are both low [7,8]. The Pan-African Society of Cardiology presented a roadmap that aims to achieve 25% hypertension control by 2025 [9]. Identifying individuals with hypertension, increasing awareness and referral to hypertension care are important first steps in improving hypertension control.

In SSA, community screening events are often held to reach and detect individuals with a disease or at risk for developing it. Hypertension screening events are also common [10]. Due to the asymptomatic nature of hypertension, affected individuals often remain undiagnosed [11]. Community screenings can potentially reach large groups of individuals and inform them on hypertension and CVD, its risk factors and accessing care. In the current literature, conflicting evidence exists on the effectiveness of community screenings. In a study from Uganda many individuals visited a healthcare provider following a community screening for hypertension [12]. However, Durão *et al*. [13] report that due to lack of studies from low- and middle-income countries there is insufficient evidence on the effectiveness of hypertension screenings.

In addition to identifying individuals at risk for CVD, improving accessibility to good-quality hypertension care is necessary to reach the 25% hypertension control target in Africa. Data from Nigeria show control rates of hypertension being between 3% and 9% [8]. Hence, a decentralized pharmacy-based hypertension care model was piloted by OMRON Healthcare in Lagos, Nigeria. Three different strategies were used to recruit hypertensive patients in the pilot program. As part of a larger study to evaluate the feasibility of the care model, we here report on our experiences with these three recruitment strategies. Elsewhere we present results of the evaluation study on patient retention, changes in blood pressure, the quality and satisfaction with the care model [14], and patients’ and healthcare provider’s perceptions and practices regarding hypertension, pharmacy-based care, and mHealth [11].

## Methods

The three strategies used to recruit hypertensive patients in the pharmacy-based hypertension care pilot program were (1) community hypertension screening events, (2) referral of hypertensive patients from Lagos University Teaching Hospital (LUTH) outpatient departments and (3) identification of individuals with elevated blood pressure at the pharmacies participating in the pilot program. The aim of the study was to describe our experiences executing each recruitment strategy, with a specific focus on the community screening events. We evaluated the community hypertension screening events using the following research questions:

1. What is the number needed to screen (NNS) to find one individual with elevated blood pressure?
2. What is the NNS to successfully enroll one individual in the pilot program?
3. What factors are associated with enrollment among those referred to the pilot program?
4. What are the most important reasons for non-enrollment in the pilot program?

### Pharmacy-based care model

The key component of the pharmacy-based care model was task-shifting from medical doctors to pharmacy staff by using a mobile application (‘mHealth app’), developed by OMRON Healthcare and their technical partners. Cardiologists remotely monitored patients accessing hypertension care at the pharmacy by review of their blood pressure data, related complaints, and drug prescriptions through the mHealth app. The pharmacist counseled the patient on drugs and lifestyle interventions, performed routine blood pressure monitoring, and dispensed drugs to the patients. Cardiologists and pharmacists were jointly responsible for remote patient monitoring and management, and communication between them was primarily through the mHealth app. Pharmacists and cardiologists received a fee for each patient monitored.

Recruited patients were registered in both the pilot program and mHealth app and were expected to stay in the pilot program for approximately six months. The patient participation fee was 250 Naira per month (≈ 0.96USD, average exchange rate May–Dec 2016), excluding the costs of medications. A more extensive description of the care model is provided elsewhere [14].

### Community screening events

**Figure 1.**
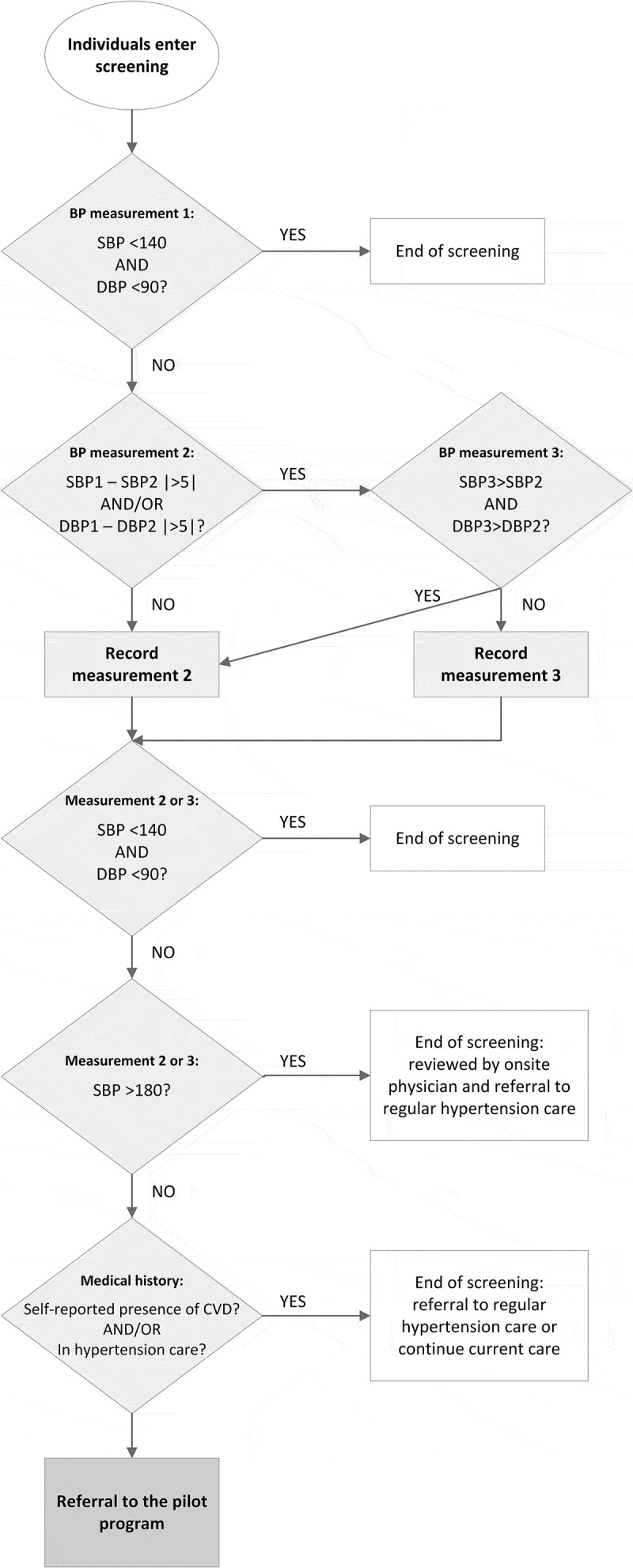
Community hypertension screening event process. The numbers refer to blood pressure values in mmHg. SBP: systolic blood pressure; DBP: diastolic blood pressure

Through community hypertension screening events, we targeted individuals living nearby the pharmacies who were not yet aware of or not yet receiving care for hypertension. Ten community screening events, two events near each participating pharmacy, were held mostly on Saturdays between February and April 2016. The events were organized by OMRON Healthcare and implementing partners. Community mobilizers actively sensitized the community about the events by engaging community leaders, advertisements, music and community volunteers. Screening staff registered individuals voluntarily attending the screening and medical doctors from LUTH measured the participant’s blood pressure after rest during registration, see Figure 1 for the screening process. Participants with elevated blood pressure (defined as systolic blood pressure (SBP) ≥140 mmHg and/or diastolic blood pressure (DBP) ≥90 mmHg) were re-measured after 1–2 minutes. For individuals with a normal blood pressure (defined as SBP <140 mmHg and DBP <90 mmHg) screening was concluded as they were deemed low-risk for hypertension and therefore not in need for care. Known hypertensives with controlled hypertension were also excluded at this stage, based on their blood pressure, as we did not want to interrupt their care. If the difference in systolic and/or diastolic blood pressure between the first and second measurement was larger than 5 mmHg, a third measurement was taken and the lower of the last two measurements was recorded. Participants with an SBP above 180 mmHg were reviewed and directly referred by the onsite medical doctor to regular care for further management of hypertensive urgency or emergency. Participants with elevated blood pressure at two measurements (the first measurement and second or third measurement), who did not self-report presence of CVD and were not currently in care with a medical doctor were eligible for referral to the pilot program. The medical doctor explained hypertension and its risk factors to all participants, if participants were eligible and expressed willingness to participate, an appointment to attend a recruitment day was made. Screening staff later reminded participants of their appointment by phone. In an event where the participant did not show up to their appointment, at least two additional phone calls were made to remind and encourage the individual to attend.

### Referral of hypertensive patients from the hospital and pharmacies

At LUTH we targeted patients visiting the cardiology, family medicine and community medicine outpatient clinics who deemed eligible for their hypertension care to be managed at the pharmacy during the pilot program, rather than at the hospital. During the recruitment period, medical doctors at these outpatient clinics were asked to identify individuals with elevated blood pressure and patients already managed for hypertension and refer those who were potentially interested in the pilot program.

Through the pharmacies participating in the pilot program, we targeted individuals who already patronized the pharmacy. These pharmacies provided free blood pressure measurement services at their customers’ request or based on presenting signs and symptoms. The pharmacists referred customers with elevated blood pressure and a potential interest in the pilot program to the recruitment days and reminded them if necessary.

### Recruitment

To be recruited into the pilot program, individuals identified with elevated blood pressure had to attend one of the recruitment days at LUTH (twice a week from February to May 2016) or one of the participating pharmacies. Initially, pharmacy-based recruitment was only planned at two pharmacies far away from LUTH, but after two weeks this was adjusted (as attendance at LUTH was low) and between 5 and 12 additional recruitment days were organized at each of the five pharmacies.

At recruitment, the pilot program’s resident cardiologists or medical doctors under supervision of a cardiologist assessed eligibility. The criteria for inclusion in the pilot program included individuals aged 18 years and above and a (new or previous) hypertension diagnosis confirmed by the cardiologist or medical doctor. Exclusion criteria were: (1) individuals with a previous history of cardiac failure, stroke or renal disease; additional risk factors for CVD identified by the cardiologist or medical doctor; individuals with an SBP ≥180 mmHg and/or DBP ≥110 mmHg were not suitable for the pilot program, as more comprehensive monitoring may be desired, which could not be guaranteed during this pilot phase; (2) individuals not permanently residing in Lagos State; and (3) pregnant women (self-reported). Individuals who were not eligible for the pilot program received lifestyle advice if applicable and were referred for regular hypertension care at a public or private health facility.

### Data collection

We collected data during both the community screening events and at the recruitment days. During screening and recruitment, blood pressure was measured on the upper left arm at heart level after at least 5 minutes of rest in a sitting position using a validated automatic blood pressure device (OMRON M6 Comfort; OMRON Corporation, Kyoto, Japan). At the community screening events, screening staff used pre-numbered duplicated record forms to organize the screening and referral process. They recorded blood pressure, age and gender for all screening participants. Additional information on the medical history, interest in the pilot program, and contact details were recorded when participants had elevated blood pressure. Screening staff provided participants with a copy of their screening results as a take-home medical record. Research staff copied data from the forms anonymously in an electronic database. For all individuals attending the recruitment days, the research staff collected data on eligibility, anthropometric and blood pressure measurements as part of the pilot program’s feasibility study.

### Statistical analysis

Data were analyzed using STATA version 12 (StataCorp LP, College Station, Texas, USA). We used multivariable logistic regression analysis corrected for clustering at the community screening event level to evaluate the association between enrollment and age, gender, blood pressure classification and hypertension status among community screening participants eligible to participate in the pilot program.

## Results

### Community screening events

**Figure 2.**
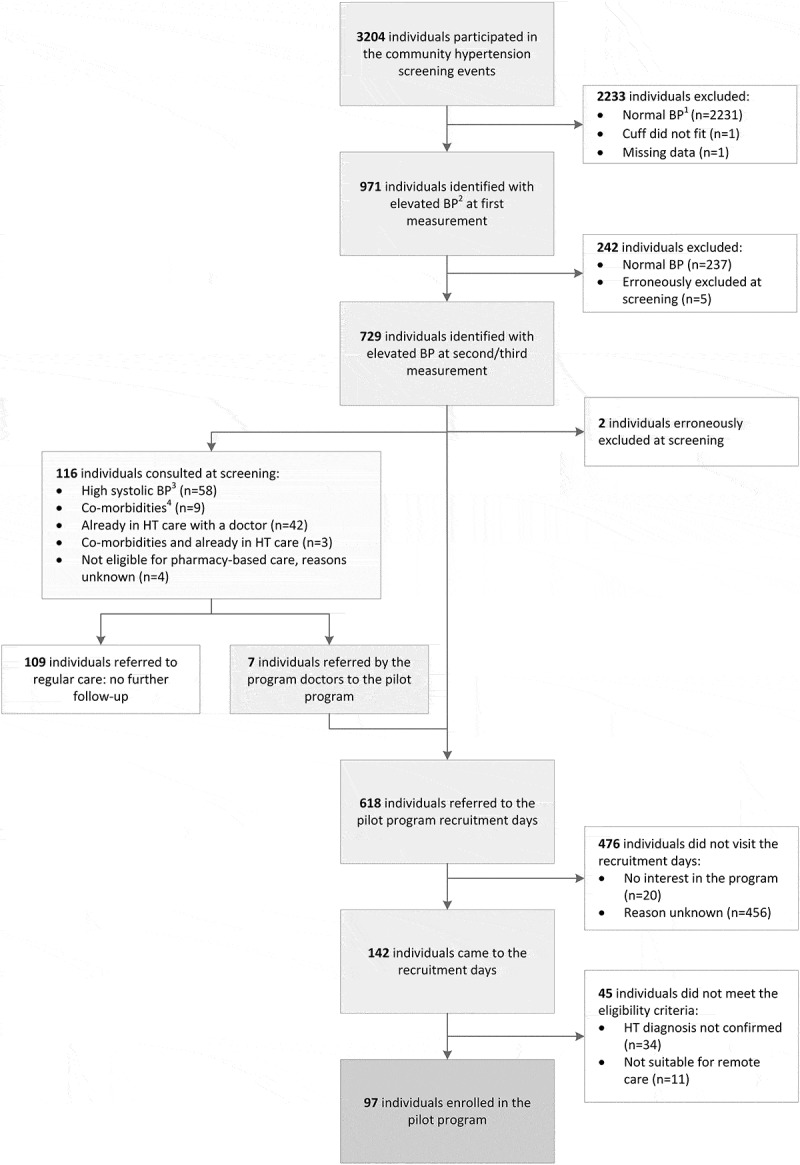
Flow chart of community hypertension screening participants (N = 3204). ^1^ A normal blood pressure was defined as a systolic BP below 140 mmHg and a diastolic BP below 90 mmHg. ^2^ Elevated blood pressure was defined as a systolic BP of 140 mmHg and above or a diastolic BP of 90 mmHg and above. ^3^ A high systolic blood pressure was defined as a systolic BP above 180 mmHg. ^4^ Individuals who were diagnosed by a doctor with cardiac failure, a stroke or kidney disease in the past were excluded from the screening. BP: blood pressure; HT: hypertension; LUTH: Lagos University Teaching Hospital

In total 3,204 individuals participated in the community screening events, of whom 729 (22.8%) had elevated blood pressure (Figure 2). The occurrence of elevated blood pressure was similar among women and men, but participants with elevated blood pressure were older (50.2 vs. 38.7 years, Table 1).

**Table 1.** Characteristics of community screening participants with a normal and elevated blood pressure (N = 3202).

	Elevated blood pressure^a^		Normal blood pressure^b^		p-value
	N		N		
**Gender, n (%)**	729		2473		0.610
Male		315 (43.2)		1095 (44.3)	
Female		414 (56.8)		1378 (55.7)	
**Age in yearsc, mean (sd)**	722	50.2 (12.7)	2458	38.7 (12.7)	<0.001
**Age in groupsc, n (%)**	722		2458		<0.001
18–24		5 (0.7)		251 (10.2)	
25–34		71 (9.8)		817 (33.2)	
35–44		171 (23.7)		672 (27.3)	
45–54		216 (29.9)		410 (16.7)	
55–64		157 (21.7)		198 (8.1)	
65–74		79 (10.9)		89 (3.6)	
≥ 75		23 (3.2)		21 (0.9)	
**Screening location, n (%)**	729		2473		0.569
Pharmacy 1		125 (17.1)		459 (18.6)	
Pharmacy 2		155 (21.3)		504 (20.4)	
Pharmacy 3		138 (18.9)		427 (17.3)	
Pharmacy 4		131 (18.0)		492 (19.9)	
Pharmacy 5		180 (24.7)		591 (23.9)	

^a^ Systolic blood pressure ≥140 or diastolic blood pressure ≥90 mmHg.

^b^ Systolic blood pressure <140 mmHg and diastolic blood pressure <90mmHg.

^c^ For 22 individuals the age was unknown.

Significant at p-value <0.05

Of the participants with elevated blood pressure, 618 (84.8%) were referred to the recruitment days of the pilot program, 109 participants (15.0%) were referred to regular hypertension care because of ineligibility or because they were already in hypertension care with a doctor, and 2 participants (0.3%) were erroneously excluded at screening (Figure 2). Of the individuals referred, 368 (59.5%) were newly diagnosed with hypertension. Among those referred, 142 participants (23.0%) visited the recruitment days, while 476 participants (77.0%) did not visit the recruitment days. Twenty participants (4.2%) indicated at the end of the community screening that they were not interested in the pilot program. For the other 456 individuals (95.8%) the reasons for not visiting the recruitment days were unclear. Ultimately 97 participants enrolled in the pilot program, 15.7% of the invited community screening participants with elevated blood pressure.

**Figure 3.**
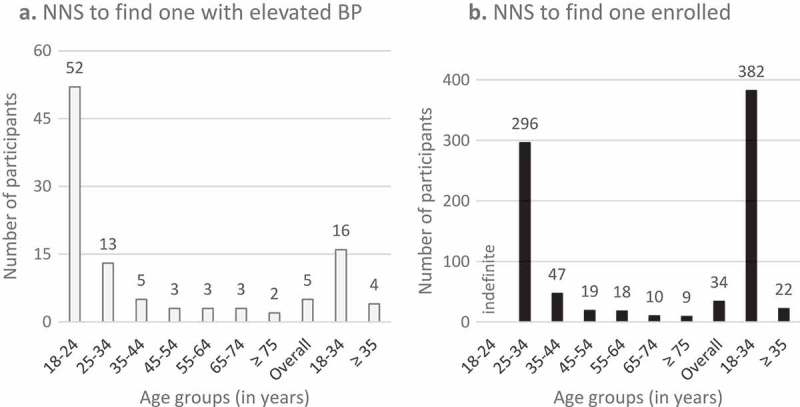
(a) Number needed to screen to find one individual with elevated blood pressure. (b) Number needed to screen to find one individual enrolled in the pilot program.

The number of individuals needed to screen (NNS) to find one individual with elevated blood pressure was 5, while the NNS was 34 to successfully enroll one individual in the pilot program. The NNS to find one individual with elevated blood pressure and to find one individual enrolled both decreased with advancing age (Figure 3). Among community screening participants aged below 35 years (36% of the population) the NNS to find one individual with elevated blood pressure was 16 compared to 4 among participants aged 35 years and above. The NNS to recruit one individual in the pilot program was 382 among participants aged below 35 compared to 22 among participants aged 35 years and above.

Forty-five participants (31.7%) who visited the recruitment days were not eligible for inclusion in the pilot program. The hypertension diagnosis was not confirmed for 34 (76%) of them, their mean SBP was 140.5 mmHg (SD 8.2) at the community screening event. Furthermore, the medical doctor deemed 11 participants (24%) not suitable for the pilot program. The main reasons being the participant did not need medication or could benefit from lifestyle treatment alone (n = 4), the blood pressure was severely high (n = 3), the participant had comorbidities (n = 2) or the participant was already in care with a medical doctor (n = 2).

Community-screening participants aged 65 and above had the highest likelihood to enroll (OR: 6.1, 95%CI: 1.5–25.0) in the pilot program compared to individuals below 35 years (Table 2). Individuals who had a blood pressure above 160/100 mmHg were more likely to enroll compared to individuals with a blood pressure between 140–159 mmHg systolic and/or 90–99 mmHg diastolic (OR: 1.7, 95%CI: 1.1–2.5). Individuals who currently use antihypertensive medication were more likely to enroll in the pilot program (OR: 2.4, 95%CI: 1.1–5.4) compared to individuals who were not diagnosed with hypertension in the past.

**Table 2.** Determinants for enrollment in the pilot program for participants who were eligible during community screening.

	Eligible to participate (N = 612^a^)	Enrolled in program, n (%) (N = 97)	Odds Ratio	95% CI
**Gender**				
Male	276	43 (15.6)	1.00	-
Female	336	54 (16.1)	1.10	(0.69–1.76)
**Age group in years**				
18–34	70	3 (4.3)	1.00	-
35–44	154	18 (11.7)	2.42	(0.88–6.67)
45–54	187	34 (18.2)	3.97	(1.13–13.93)
55–64	123	20 (16.3)	3.41	(1.22–9.51)
≥ 65	78	22 (28.8)	6.13	(1.50–24.98)
**Blood pressure classification JNC7**				
Stage 1: SBP 140–150/DBP 90–99	397	51 (12.8)	1.00	-
Stage 2: SBP ≥ 160/≥ 100	215	46 (21.4)	1.67	(1.12–2.49)
**Hypertension status**				
Not diagnosed in the past	364	44 (12.1)	1.00	-
Diagnosed in the past, but not taking antiHT medication	155	26 (21.4)	1.34	(0.80–2.26)
Diagnosed in the past and currently taking antiHT medication	93	27 (29.0)	2.42	(1.09–5.39)
**Pharmacy**				
Pharmacy 1	112	16 (14.3)	1.00	-
Pharmacy 2	132	23 (17.4)	1.34	(0.63–2.84)
Pharmacy 3	119	13 (10.9)	0.77	(0.33–1.79)
Pharmacy 4	114	27 (23.7)	2.19	(1.25–3.83)
Pharmacy 5	135	18 (13.3)	1.03	(0.53–2.02)

^a^ Of the 618 eligible participants, 6 individuals have missing data on age and are not included in the analysis.

SBP: systolic blood pressure; DBP: diastolic blood pressure; HT: hypertension

### Hospital and pharmacies

Three individuals were referred for recruitment from the LUTH outpatient clinics compared to 258 referred from pharmacies. Eventually, 2 individuals referred from LUTH and 226 individuals (87.6%) referred from the pharmacies enrolled in the pilot program. Of the individuals referred from the pharmacies, 29 (13%) were newly diagnosed with hypertension. In total, 33 individuals were not eligible to participate, 20 of which were not confirmed to be hypertensive by the medical doctor. For 11 of the individuals, the medical doctor considered the pilot program not suitable, the main reasons being: the patient did not need medication (n = 2), the blood pressure was severely high (n = 3), the patient had comorbidities (n = 5) or the patient was already in care (n = 1). The reason was unknown for the remaining two individuals.

An additional three participants who were seen at the screening events, but initially not eligible, later enrolled in the pilot program via the pharmacies. In total 328 patients enrolled in the pilot program, 69% via pharmacy referral, 30% through community screening events, and less than 1% via referral of the LUTH outpatient clinics.

## Discussion

This study was part of a larger study investigating the feasibility of a pharmacy-based hypertension care model that included an mHealth app for remote patient monitoring by cardiologists in Lagos [11,14]. The lessons learned from this study are that community screening events appeared successful in identifying individuals with elevated blood pressure. Second, referral through pharmacies appeared more effective than referral from community screening events in recruiting patients. Third, referral of hypertensive patients from a tertiary hospital to community pharmacies appeared challenging.

Although we found a high proportion of individuals with elevated blood pressure during the community screening events (NNS = 5), this was lower compared to previous studies in Lagos [1–3]. The prevalence of hypertension in Lagos is high [1,2] and we expected a high yield of participants for the pilot program through the community screening events. However, recruitment in the pilot program was lower than expected, suggesting that the screening events did not attract the intended population. The young age of the participants contributed to the low effectiveness of the screening events. Similar to another screening event organized in Lagos [1], we screened many individuals aged below 35 years, among whom the NNS to find one individual with elevated blood pressure was 4 times higher compared to older participants (16 vs. 4), and the likelihood to enroll in the pilot program lower. Restricting screening to individuals aged 35 years and above would have reduced the screening load by 36% but would have reduced enrollment in the program by 3% only.

Furthermore, the screening events attracted a substantial proportion of individuals who appeared already aware of their condition and on antihypertensive medication. These participants were more likely to enroll in the pilot program compared to those unaware of their hypertension status or not yet in care. However, we had anticipated to identify the latter groups. A study into the reasons why individuals participate in health events such as community screenings would therefore be worthwhile.

Low attendance on the recruitment days following the community screening events, despite several attempts to reach individuals by phone. This may have been because screening participants did not have a prior relationship with the pharmacist, as opposed to referrals from the pharmacy. Our qualitative research showed that the relationship with the pharmacist is an important factor in healthcare-seeking behavior and retention in care [11]. During reflections with the pharmacists, they additionally suggested that community screening participants may have been more inclined to attend the recruitment days if the involvement and visibility of the pharmacy in the community screening events had been larger. For example, the logo of Nigerian parties including the pharmacies was not shown on the flyers and posters, giving participants the idea that the pilot program was foreign-run, instead of run by the pharmacies. The importance of a personal connection with the pharmacist may also explain that referral through the pharmacies was most successful in recruiting hypertensive patients in the pilot program. Possibly for individuals who already accessed (hypertension care) services at the pharmacy, the recruitment days may have felt more accessible. However, there was no difference in retention in the pilot program between those referred via the pharmacies compared to through community screening events [14].

Low numbers of individuals utilizing healthcare providers after community and home-based screening events was also observed in other sub-Saharan African settings [15–18]. Some of the reasons for this low attendance include individual (e.g. asymptomatic nature of hypertension, financial constraints), environmental (e.g. distance), socioeconomic (e.g. poverty), and health system factors (e.g. popularity of alternative therapies) [19]. In a study from Uganda, where they implemented an intervention that included a transport voucher to a health facility to enhance healthcare seeking, a very high proportion of individuals attended a healthcare provider for hypertension (83%) within six months after a community-based screening [12]. The lack of a transport reimbursement, or long distance to the health facility seem unlikely reasons why our community screening participants did not attend the recruitment days, since the events targeted neighborhoods nearby the pharmacies. The monthly participation fee may have been a barrier, however the fee was small and included a free first visit to the medical doctor.

Regarding the third lesson, the reasons why referral via LUTH was not successful in recruiting patients in the pilot program included conflict of interest, as the departments needed to reach their patient targets, medical doctors being on strike, and potentially eligible patients did not live near the pharmacies (personal communication).

The lessons learned from the recruitment strategies cannot be generalized to recruitment in regular hypertension care as patients were recruited for a specific pharmacy-based pilot program with its own specifications and individuals needed to be eligible for the pilot program. One of the strengths of this evaluation is that we used individual level data obtained at several community screening events. A limitation of our evaluation is that we were unable to collect the number of people approached at LUTH and the pharmacies for referral to the pilot program, but only collected data of individuals who visited the recruitment days. Nevertheless, the available data provide lessons for future recruitment activities, suggesting that if decentralized pharmacy hypertension care is implemented in comparable settings, recruitment could focus primarily on pharmacy clientele. Our study confirms the limited value of community hypertension screening events in this setting.

## Conclusion

We found that pharmacies are essential in the recruitment of hypertensive patients in a pharmacy-based hypertension care pilot program. For future programs we recommend the use pharmacy referral as individuals are more inclined to access care through healthcare providers that they are familiar with. Recruitment through community screening events and a hospital’s outpatient clinics resulted in a lower number of recruited patients than expected. More research is needed to better understand why individuals newly identified with hypertension do not access care, as different approaches may be needed to enroll them in care.

## Paper context

In sub-Saharan Africa community screenings are often organized for different diseases and symptoms while their effectiveness is debated in the literature. To the best of our knowledge, this is one of the first studies that compare different strategies, including community screenings, to recruit individuals in pharmacy-based hypertension care. Despite the potential attractiveness of community screenings, we experienced that individuals seem more inclined to access care through the pharmacists, i.e. healthcare providers they are familiar with.
